# Associations between treatment credibility, patient expectancies, working alliance and symptom trajectory in cognitive behaviour therapy for pathological health anxiety

**DOI:** 10.1111/papt.12591

**Published:** 2025-03-27

**Authors:** Erland Axelsson, Erik Hedman‐Lagerlöf

**Affiliations:** ^1^ Division of Family Medicine and Primary Care, Department of Neurobiology, Care Sciences and Society Karolinska Institutet Stockholm Sweden; ^2^ Liljeholmen University Primary Health Care Centre, Academic Primary Health Care Centre Region Stockholm Stockholm Sweden; ^3^ Division of Psychology, Department of Clinical Neuroscience Karolinska Institutet Stockholm Sweden; ^4^ Gustavsberg University Primary Health Care Centre, Academic Primary Health Care Centre Region Stockholm Stockholm Sweden

**Keywords:** cognitive behaviour therapy, expectations, health anxiety, illness anxiety disorder, somatic symptom disorder, therapeutic alliance

## Abstract

**Objectives:**

To evaluate how treatment credibility, the expectancy of improvement and the relationship with the therapist (the working alliance) change in relation to symptoms in cognitive behaviour therapy (CBT) for pathological health anxiety.

**Design:**

Secondary study of a randomised controlled trial of Internet‐delivered (*n* = 102) and face‐to‐face CBT (*n* = 102) for health anxiety.

**Methods:**

The trial was conducted at a primary health care clinic in Stockholm, Sweden, between December 2014 and July 2018. Both treatments lasted 12 weeks. Health anxiety was measured using the 18‐item Health Anxiety Inventory. Credibility/expectancy (Borkovec credibility/expectancy scale) and the strength of the working alliance (Working Alliance Inventory) were self‐reported by the participant at weeks two and eight. Symptom slopes from a linear mixed model were related to these process scales.

**Results:**

Correlations between the process variables (credibility/expectancy, working alliance) and the overall, 12‐week pre‐ to post‐treatment, reduction in health anxiety were small to moderate, and slightly higher based on data from week 8 (*r*s = 0.33–0.41) than week 2 (*r*s = 0.17–0.29). In the whole sample, week 2 credibility/expectancy and working alliance were significant predictors of subsequent symptom reduction. In secondary subgroup analyses, the process variables predicted improvement in Internet‐delivered CBT, but not in face‐to‐face CBT. Direct between‐format tests were not significant. Week 8 credibility/expectancy and working alliance were more closely related to previous than subsequent symptom reduction.

**Conclusions:**

The patient's early ratings of credibility/expectancy and the strength of the working alliance appear to be predictive of subsequent symptom reduction. Later ratings appear to be of more limited predictive utility.

## INTRODUCTION

Pathological health anxiety is characterised by a recurrent and excessive fear of, or preoccupation with, having or developing a serious disease such as terminal cancer, a cardiovascular disease or a neurological disorder (Asmundson et al., 2012; Ferguson, 2009; Kellner, 1986; Longley et al., 2010; Salkovskis et al., 2002). This is typically coupled with a pronounced focus on bodily processes and a strong fear of death (Aan de Stegge et al., 2018; Höfling & Weck, 2013). Depending on the precise definition, pathological health anxiety has a prevalence of approximately 0.4%–13.1%, with most estimates around a few percent (Weck et al., 2014). The condition is associated with increased functional impairment and healthcare costs (Sunderland et al., 2013) and also adverse health outcomes (Mataix‐Cols et al., 2024). Approximately two‐thirds of patients respond to cognitive behaviour therapy (CBT), which can be delivered in many formats, including via the Internet and as a traditional face‐to‐face treatment (Axelsson & Hedman‐Lagerlöf, 2019; Cooper et al., 2017). Little, however, is known about the processes that contribute to this treatment effect. At least some of the beneficial effects of CBT for health anxiety would be expected to be the result of *common factors*, that is mechanisms common to all, or at least most, psychotherapies (Cuijpers et al., 2019; Kirsch, 2005).

One such common factor is the degree to which the patient finds the treatment credible and expects future improvement (Bressan et al., 2017; Friedman, 1963). In approaching this question, a distinction is often made between *treatment credibility*, which refers to ‘how believable, convincing, and logical the treatment is’, and *expectancy*, which refers to ‘improvements that clients believe will be achieved’ (Kazdin, 1979). Credibility and the expectancy of improvement can moderate both the effect, and the tolerability, of clinical interventions (Colloca & Miller, 2011; Kaptchuk & Miller, 2015). In psychotherapy, less is certain, however, about in precisely what manner and to what extent. A recent systematic review based on 27 studies reported a weak but significant pooled correlation (*r* = 0.15) between treatment credibility and outcome (Kumpasoğlu et al., 2024). Tentatively, based on a recent meta‐analysis of randomised controlled trials, treatment credibility and the expectancy of improvement appears to be about as important for the overall treatment effect in Internet‐delivered as in face‐to‐face CBT (Pontén et al., 2024). In a systematic review published in 2023, treatment credibility was also identified as one of only three consistent predictors of treatment outcome in therapist‐guided Internet‐delivered treatments (Haller et al., 2023). In line with this finding, treatment credibility and expectancy has been found to be predictive of efficacy also in Internet‐delivered CBT for pathological health anxiety specifically (Hedman et al., 2015). Moreover, lower credibility/expectancy ratings early in treatment have been found to predict later dropout (Axelsson & Hedman‐Lagerlöf, 2023a). It is unclear, however, how credibility/expectancy and symptom levels interact over time in CBT for health anxiety.

Most psychological treatments also involve a therapist. It is well established that a collaborative environment between the therapist and the patient—one where an emotional bond is coupled with an agreement on treatment goals and how to approach key processes—is typically predictive of better outcomes. This combination of an emotional bond and the agreement on goals and processes is commonly referred to as the *working alliance* (Bordin, 1979; Elvins & Green, 2008; Fluckiger et al., 2018; Wampold & Fluckiger, 2023). Baier et al. (2020) surveyed previous meta‐analyses and found that these reported relatively consistent pooled overall correlations between alliance and outcome, in the weak to lower‐moderate range (*r* = 0.21–0.28) (Fluckiger et al., 2018; Horvath et al., 2011; Horvath & Symonds, 1991; Martin et al., 2000). The therapeutic alliance has also been found to mediate psychotherapy outcomes in 70% of existing original studies where this was tested (Baier et al., 2020). Studies have indicated that this is relevant also for “hands‐on” CBT such as therapies focusing on exposure and response prevention in the treatment of anxiety and obsessive‐compulsive spectrum disorders (Buchholz & Abramowitz, 2020; Strappini et al., 2022). Recent advances in modelling strategies and the recruitment of larger sample sizes in clinical psychology have enabled the study of more ‘state‐like’ (i.e. short term, within‐individual) interactions between the strength of the working alliance and treatment outcome over the course of psychotherapy. An individual participant data meta‐analysis published in 2020 found, based on 17 primary studies with 5350 participants, that there is typically a reciprocal relationship between the strength of the working alliance and the main clinical outcome, at least in the first weeks of treatment (Fluckiger et al., 2020). This is to say that, over the initial course of treatment, whenever the working alliance is strengthened, this typically contributes to subsequent symptom reduction and vice versa. Whether or not such findings should be expected to generalise to all forms of psychotherapy to an equal extent is contested (Baier et al., 2020). Focusing on face‐to‐face CBT for pathological health anxiety specifically, the working alliance has been found to be predictive of the clinical outcome in at least one clinical trial (Weck et al., 2015). For Internet‐delivered psychological treatments in general, a recent meta‐analysis indicated that the pooled overall correlation between the working alliance and the efficacy of the treatment was weak but significant (*r* = 0.20) (Kaiser et al., 2021). Similar to treatment credibility, the working alliance has been identified as one of three consistent predictors of outcome in therapist‐guided Internet‐delivered therapies (Haller et al., 2023) and similar results have been documented with regard to Internet‐delivered CBT for pathological health anxiety specifically (Axelsson & Hedman‐Lagerlöf, 2023a; Hedman et al., 2015). Little is known, however, about the reciprocal interplay between the working alliance and symptom levels over time in CBT for health anxiety.

The overarching aim of the present study was to model how treatment credibility, the expectancy of improvement and the strength of the working alliance as rated by the patient develop over time and interact with symptom trajectories in Internet‐delivered and face‐to‐face CBT for pathological health anxiety. Based on the available evidence base and mainstream opinion of the field (see above), the expectation was that of a reciprocal relationship between credibility/expectancy and working alliance on the one hand, and the reduction in health anxiety on the other. This study focused on between‐individual variance, that is whether individuals with comparably high credibility/expectancy and working alliance ratings would report more symptom reduction than other individuals, and whether individuals who reported more symptom reduction would then have higher credibility/expectancy and working alliance ratings than other individuals.

## METHODS

### Study design

This was a secondary analysis of data from a randomised controlled non‐inferiority trial of Internet‐delivered versus face‐to‐face CBT for pathological health anxiety (*N* = 204) (Axelsson et al., 2020), based at Gustavsberg University Primary Health Care Center and conducted in collaboration with Karolinska Institutet, Stockholm, Sweden. The trial was designed for 80% power to confirm non‐inferiority of Internet‐delivered relative to face‐to‐face CBT in the primary analysis of efficacy (see the primary publication above). The protocol was preregistered at ClinicalTrials.gov (NCT02314065), and the statistical analysis plan for this secondary study was registered in the Open Science Framework (b785z) before the corresponding analyses were conducted. All results are reported in accordance with the CONSORT 2010 guidelines (Schulz et al., 2010).

### Participants

This trial employed a mixed recruitment strategy, where participants were recruited both via advertisement (primarily in local newspapers under the heading ‘Do you worry a lot about your health?’; a key item of the Whiteley Index [Pilowsky, 1967]) and routine care (the study site and also referrals from other primary care and psychiatric clinics in Stockholm). Information about the trial stated clearly that the treatment was intended for individuals with ‘a persistent fear of being ill or becoming ill’. The study website was open for applications from both pathways, where each applicant first provided informed consent, before completing an online screening battery primarily based on self‐report questionnaires. Applicants subsequently underwent a face‐to‐face eligibility interview with a clinical psychologist at the study site. This interview was also used as an opportunity to collect additional clinical data, primarily about psychiatric diagnoses including DSM‐IV hypochondriasis as assessed using the Anxiety Disorders Interview Schedule for DSM‐IV (DiNardo et al., 1994), and to discuss what it would mean to take part in exposure‐based CBT for health anxiety (Hedman‐Lagerlöf & Axelsson, 2019). Adults (18+ years) living in Stockholm who met full criteria for a primary diagnosis of DSM‐5 somatic symptom disorder or illness anxiety disorder as assessed using the Health Preoccupation Diagnostic Interview (Axelsson et al., 2016) in conjunction with the Mini‐International Neuropsychiatric Interview (Sheehan et al., 1998) were included in the trial. Following removal of the hypochondriasis diagnosis, somatic symptom disorder and illness anxiety disorder are the prototypical diagnoses for pathological health anxiety in the DSM‐5 (Dimsdale et al., 2013). In broad terms, a diagnosis of somatic symptom disorder is typically made when the patient's health anxiety is related to somatic symptoms, whereas a diagnosis of illness anxiety disorder is typically made when there are no or only mild somatic symptoms (American Psychiatric Association, 2022). Tentatively, making this distinction between somatic symptom disorder and illness anxiety disorder appears to be relatively unimportant for the effects of CBT when the patient suffers from pathological health anxiety as defined in the introduction, and when the treatment focuses on this problem (Axelsson & Hedman‐Lagerlöf, 2023b; Newby et al., 2017; Newby et al., 2018). Exclusion criteria were: another primary psychiatric disorder (comorbidities were allowed), a substance or alcohol use disorder during the past six months, a psychotic or bipolar disorder, severe depression or recurrent suicidal ideation, concurrent psychological treatment, CBT for health anxiety in the past year, and a serious somatic disorder such as terminal cancer or a severe neurological condition. Participants were also required to either not be on antidepressants, or to express an intent to maintain a stable dosage throughout the main phase of the trial.

**FIGURE 1:**
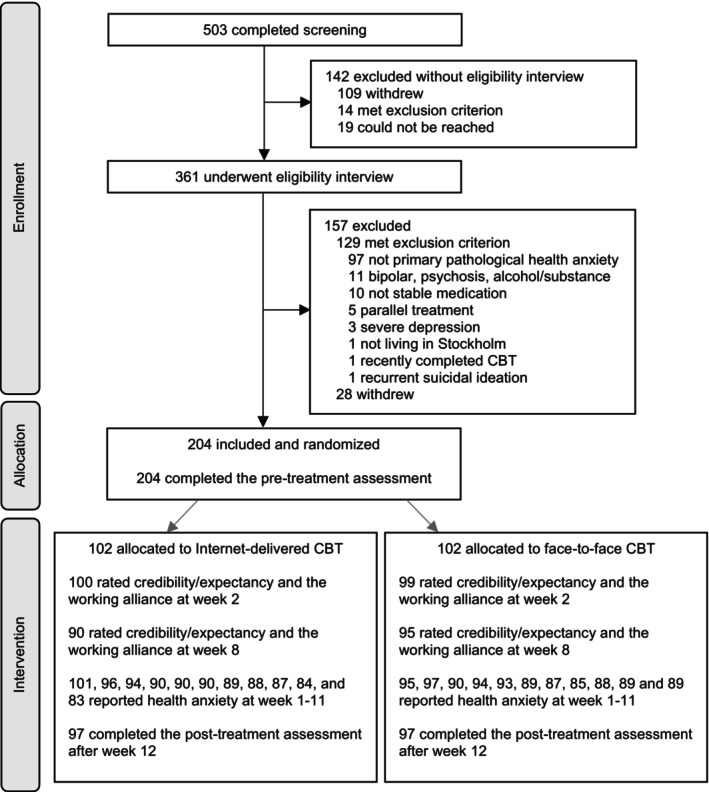
Participant flow including rates of missing data in this randomised controlled trial of therapist‐guided Internet‐delivered versus face‐to‐face cognitive behaviour therapy for pathological health anxiety. CBT, cognitive behaviour therapy.

The study flow is illustrated in Figure 1. Most participants (143/204, 70%) were female and the mean age was 39 (SD = 12, range 18–78). The majority of the sample had suffered from pathological health anxiety for several years (M = 9.2, SD = 9.4), and about every third participant (67/204) was recruited via the routine care environment (either the study site, or via referral from other clinics in Stockholm). See Table 1 for more sample characteristics.

**TABLE 1:** Sample characteristics.

Variable	Estimate	Internet‐delivered CBT (*n* = 102)	Face‐to‐face CBT (*n* = 102)
		*n* (%)/*M* (*SD*), range	*n* (%)/*M* (*SD*), range
Age		39 (12), 19–65	39 (13), 18–78
Gender	Female	72 (71%)	71 (70%)
Educational attainment	Below upper secondary	2 (2%)	3 (3%)
	Upper secondary	24 (24%)	21 (21%)
	Tertiary	76 (75%)	78 (76%)
Employment	Working full time	60 (59%)	50 (49%)
	Working part time	12 (12%)	15 (15%)
	Student	12 (12%)	10 (10%)
	Retired	3 (3%)	7 (7%)
	Unemployed	3 (3%)	4 (4%)
	Other	12 (12%)	16 (16%)
Years with pathological health anxiety		9.3 (10.4), 0.5–60	9.1 (8.5), 0.5–36
Health anxiety (HAI‐18^a^)		33.9 (6.5), 15–48	34.2 (6.4), 20–51
Diagnosis according to the DSM‐5	Somatic symptom disorder	58 (57%)	58 (57%)
	Illness anxiety disorder	46 (45%)	44 (43%)
Diagnosis according to the DSM‐IV	Hypochondriasis	98 (96%)	93 (91%)
	Not hypochondriasis	4 (4%)	9 (9%)
Afraid that may have cancer	Most of the time	37 (36%)	45 (44%)
	Often	39 (38%)	40 (39%)
	Sometimes	20 (20%)	13 (13%)
Afraid that may have heart disease	Most of the time	12 (12%)	13 (13%)
	Often	22 (22%)	15 (15%)
	Sometimes	28 (27%)	38 (37%)
Afraid that may have other serious illness	Most of the time	18 (18%)	31 (30%)
	Often	42 (41%)	35 (34%)
	Sometimes	32 (31%)	15 (15%)
Fear of death	Most of the time	55 (54%)	54 (53%)
	Often	22 (22%)	22 (22%)
	Sometimes	20 (20%)	23 (23%)
Probable current cyberchondriasis		46 (45%)	48 (47%)
Probable health anxiety by proxy	Among parents only	39/57 (68%)	45/70 (64%)
Comorbid anxiety disorder or OCD^b^	At least one	67 (66%)	59 (58%)
Comorbid major depressive disorder		23 (23%)	21 (21%)
Depression symptoms (MADRS‐S^c^)		13.7 (6.9), 0–34	14.7 (7.0), 1–36
Disability (SDS^d^)		11.4 (7.5), 0–27	11.6 (6.8), 0–26
Path of recruitment	Routine care	39 (38%)	28 (27%)
	Other pathways	62 (62%)	74 (73%)

*Note*: ‘Probable current cyberchondriasis’ implies a response of at least ‘Agree mostly’ to the statement ‘I seek information from the Internet or in books about different illnesses that I am afraid of being afflicted by’. ‘Fear of cancer’ refers to item #16 of the Illness Attitude Scales which is phrased ‘Are you afraid that you may have cancer?’. ‘Fear of heart disease’ refers to item #17 of the Illness Attitude Scales which is phrased ‘Are you afraid that you may have heart disease?’. ‘Fear of other serious illness’ refers to item #18 of the Illness Attitude Scales which is phrased ‘Are you afraid that you may have another serious illness?’. A follow‐up free‐text item asked about which illness. Of those 181 who did not answer ‘no’ to item #18, 68 (38%; meaning 68/204 = 33% of the total sample) mentioned a fear of having a neurological disorder. ‘Fear of death’ refers to item #14 of the Illness Attitude Scales which is phrased ‘Does the thought of death scare you?’. ‘Probable health anxiety by proxy’, reported for parents only, implies a response of at least ‘Agree mostly’ either to the statement ‘I check up on my relatives' health’ or the item ‘If I notice a bodily symptom in a relative, I try to convince this person to seek health care’.

^a^ HAI‐18 = 18‐item Health Anxiety Inventory.

^b^ MADRS‐S = self‐report version of the Montgomery‐Åsberg Depression Rating Scale.

^c^ OCD = obsessive‐compulsive disorder.

^d^ SDS = Sheehan Disability Scale.

### Procedure

After the eligibility interview, those who met all eligibility criteria and completed the pre‐treatment assessment were randomised 1:1 to therapist‐guided Internet‐delivered CBT (*n* = 102) or face‐to‐face CBT (*n* = 102). This was done by an individual otherwise not involved in the trial, using a true random number service (www.random.org) and in consecutive even‐numbered cohorts. Recruitment ended when the target sample size of 200 had been met, and scheduled interviews had been completed. Because the rationale for the trial was to determine if online treatment was non‐inferior to the face‐to‐face format, both therapies were based on the same protocol originally developed for DSM‐IV hypochondriasis. Participants in Internet‐delivered and face‐to‐face CBT were all enrolled in treatment in the routine clinical setting and were presented with the same theoretical rationales, behavioural strategies, introductions to tailored homework exercises, equivalent worksheets and so on. As is commonplace in CBT for pathological health anxiety (Axelsson & Hedman‐Lagerlöf, 2019), the treatment was primarily based on exposure and response prevention (Furer & Walker, 2005). This means that patients were encouraged to engage in situations that gave rise to health anxiety while abstaining from strategies to reduce health anxiety in the short term in order to achieve therapeutic effects. For example, a participant with a fear of reading about cancer would typically be encouraged to repeatedly do so while refraining from strategies to reduce anxiety such as repeatedly asking family members for reassurance. Importantly, the treatment was tailored for the individual on the basis of functional analysis. In addition to exposure, the treatment also involved mindfulness training as a means of increasing the patient's willingness to experience anxiety. CBT for health anxiety is an active form of treatment, where the patient is expected to work with adapted homework exercises on a daily basis (Hedman‐Lagerlöf & Axelsson, 2019). Face‐to‐face CBT was provided over 12 approximately weekly sessions. The first session was scheduled to last 80 min, and subsequent sessions lasted up to 50 min. A relatively extensive package of written information with worksheets and examples was provided as supportive material between sessions. Internet‐delivered CBT was provided in a therapist‐guided text‐based format, where the content was divided into 12 modules, reminiscent of book chapters, presented at the treatment web platform with images, worksheets and weekly questions for reflection and feedback. Participants in Internet‐delivered CBT were encouraged to work relatively independently with their treatment but also communicated regularly with a therapist via an asynchronous email‐like system. Communication could be initiated either by the participant or the therapist. Most feedback by the therapist was brief emotional support and guidance in response to the weekly questions for reflection, which could be completed by the participant at any point in time to signal willingness to proceed to the next module. Whenever such questions had been completed, or the participant had written a free‐text message, the therapist would reply within two workdays. In cases where participants showed inactivity over more than a few days, the therapist was instructed to telephone the patient to promote adherence, but this was not used as the primary method of conveying the treatment principles. Participants were encouraged to complete about one module per week, and access to new modules was given sequentially by the therapist to the extent that previous modules were completed. The same therapists provided Internet‐delivered and face‐to‐face CBT and were clinical psychologists with training in CBT who worked under the supervision of experts. For more details about treatments and therapists, see the primary publication (Axelsson et al., 2020).

### Outcomes

Health anxiety was measured using the 18‐item Health Anxiety Inventory (HAI‐18) (Salkovskis et al., 2002) before treatment, each week during treatment, and again after treatment—that is with 13 measurement points in total over the treatment phase. Each item of the HAI‐18 requires the respondent to choose the most accurate among four statements, for example from ‘I do not worry about my health’ to ‘I spend most of my time worrying about my health’. The HAI‐18 has typically been found to exhibit adequate psychometric properties (Alberts et al., 2013) and can be scored either as a full‐scale sum or in terms of its two, or possibly three, subfactors (Salkovskis et al., 2002). Each item is scored 0–3, which results in a theoretical range of 0–54. In a psychiatric setting, a cut‐off on the HAI‐18 to screen for pathological health anxiety with equally weighted sensitivity and specificity is likely to approximate a score of ca 26–27 (Axelsson et al., 2023). It should be noted, however, that individuals with pathological health anxiety can also score considerably lower (Österman et al., 2022). In the pathological symptom range, mild symptoms are observed up to ca 33–34, and moderate symptoms to ca 40–41, above which levels are substantial even within the pathological range (Axelsson et al., 2023).

Credibility/expectancy was measured using the Credibility/Expectancy (C/E) scale (Borkovec & Nau, 1972) administered at week two and eight. The C/E scale is among the most widespread measures of credibility and expectancy. This version comprised the following five items: (1) ‘How logical does this type of treatment seem to you?’, (2) ‘How confident are you that this method will be successful in the treatment of your health condition?’, (3) ‘How confident would you be in recommending this treatment method to a friend with the same type of problem that you have?’, (4) ‘How successful do you think that this treatment would be in the treatment of other fears?’ and (5) ‘How much do you expect to improve from this treatment?’. Each item was scored 0–10 and the scale was summed 0–50. This unifactorial scoring has been used in previous projects (Hedman et al., 2014; Hedman et al., 2016) and was corroborated by factor analysis in the present study (results).

The strength of the working alliance was measured using a 6‐item version of the Working Alliance Inventory (WAI) (Hatcher & Gillaspy, 2006) administered at week two and eight. The WAI was originally developed based on the theoretical approach of Bordin (1979), according to which the alliance combines an emotional bond with agreement on goals and tasks. The items of the WAI are phrased in a manner neutral to the treatment framework. The following items were selected for a previous clinical trial as based on strong correlations with the sum score of a 12‐item version (Hedman et al., 2014): (1) ‘My therapist and I agree on what I have to do in treatment to improve my situation’, (2) ‘I trust that my therapist is able to help me’, (3) ‘My therapist and I work towards mutually agreed upon goals’, (4) ‘I feel that my therapist appreciates me’, (5) ‘We agree on what is important for me to work on’ and (6) ‘We have arrived at a shared understanding of the kind of changes that would be good for me’. Each item was scored 1–7, which resulted in a full scale sum score between 6 and 42.

Sociodemographic (e.g., age, gender) and key clinical data (e.g., treatment preference) were collected during the eligibility interview. To characterise the sample and also to account for pre‐treatment characteristics in the regression models (see below), this study was also based on data derived from the following measures administered at the pre‐treatment assessment: the self‐report version of the Montgomery‐Åsberg Depression Rating Scale (MADRS‐S) as a measure of depression symptoms (Svanborg & Åsberg, 1994), the 16‐item Anxiety Sensitivity Index (ASI) as a measure of anxiety sensitivity (Reiss et al., 1986) and the Sheehan Disability Scale (SDS) as a measure of disability (Leon et al., 1997). Specific items were also derived from the Illness Attitude Scales (Kellner, 1986). Adherence was quantified in terms of the number of modules in Internet‐delivered CBT and the number of attended sessions in face‐to‐face CBT. In this trial, having initiated at least six modules or sessions was the preregistered criterion for treatment completion. Several other outcomes were also collected as part of the trial and are reported elsewhere (Axelsson et al., 2020).

### Statistical analysis

Analyses were conducted in Stata 15.1 (data curation and piecewise regression model) and R 4.3.2 with mice 3.16.0 and nlme 3.1–164 (imputation and subsequent analyses), and, in accordance with a preregistered statistical analysis plan (Open Science Framework identifier: b785z). We modelled change in health anxiety (HAI‐18) over the 13 measurement points of the 12‐week treatment period using linear mixed effects regression. Because all 204 participants completed the pre‐treatment assessment, all were included in the analysis. The data was organised in long format, with three distinct time variables. This enabled the linear regression model to capture distinct rates of change before and after administration of the process scales at week 2 and 8. One time variable captured change from week 0 to 2. This variable was coded 0 at week 0, 1 at week 1, and 2 at all subsequent timepoints. A second time variable captured change from week 2 to 8. This was coded 0 up to week 2, 1 at week 3, 2 at week 4, 3 at week 5, 4 at week 6, 5 at week 7, and 6 at subsequent timepoints. A third time variable captured change from week 8 to 12. This was coded 0 up to week 8, 1 at week 9, 2 at week 10, 3 at week 11, and 4 at week 12. Random effects were added for each of these variables (slopes). In total, the linear mixed effects regression model had four random effects: the random intercept, the random effect of time from week 0 to week 2, the random effect of time from week 2 to week 8, and the random effect of time from week 8 to week 12. These three random slopes were necessary to capture change on the level of individuals, implied clearly improved model fit as based on the AIC and BIC, and thereby enabled subsequent analyses as detailed below. See the supplement for the regression equation. For each time period, change scores were derived from the fitted regression lines.

Before proceeding with further analyses, we conducted a factor analysis of the C/E scale at week 2 to determine if it would be most appropriate to analyse credibility/expectancy as one composite variable (sum score), or more correct to regard credibility and expectancy as distinct latent constructs (subscales). This was based on principal axis factoring and visual inspection of the scree plot.

In order to enable intention‐to‐treat analysis of the credibility/expectancy and working alliance outcomes, we employed multiple imputation by chained equations of 20 datasets based on predictive mean matching. In accordance with best‐practice recommendations, separate imputation models were run for Internet‐delivered and face‐to‐face CBT in order to preserve condition‐specific relationships in the data (Schafer & Graham, 2002). Subsequent analyses were then run on the multiply imputed data.

We employed *t*‐tests of differences in mean credibility/expectancy and working alliance between Internet‐delivered and face‐to‐face CBT at weeks 2 and 8. Separate linear regression models were then fitted for credibility/expectancy and the strength of the working alliance: First, change in health anxiety from week 2 to 8 (i.e. the fitted slope from the linear mixed model; see above) was regressed on the process score (C/E scale or WAI) at week 2. Second, the process score at week 8 was regressed on the process score at week 2 and the change in health anxiety from week 2 to 8. Third, change in health anxiety from week 8 to 12 was regressed on change in health anxiety from week 2 to 8 and the process score at week 8. Each model was first fitted on the entire dataset and then separately for Internet‐delivered and face‐to‐face CBT. In auxiliary analyses, we entered treatment format as an independent variable in the full data models and conducted direct tests of whether the treatment format moderated the effect of the process score on subsequent symptom change. Standardised coefficients are reported as *t*‐scores. For tests where change on the HAI‐18 was the dependent variable, we also report the incremental Cohen's *d*, that is the estimated mean difference for every increase of 1 in the independent variable, standardised on the HAI‐18 pre‐treatment standard deviation, which was 6.4.

## RESULTS

### Adherence to cognitive behaviour therapy

The participants in Internet‐delivered CBT initiated a mean of 9.0 modules (*SD* = 3.4, median = 11), and the participants in face‐to‐face CBT completed a mean of 10.6 sessions (*SD* = 3.0, median = 12). Eighty‐one participants in Internet‐delivered CBT (79%) and 92 participants in face‐to‐face CBT (90%) initiated at least six modules or sessions, which was the preregistered criterion for treatment completion.

### Factor analysis of credibility/expectancy scale

Data was suitable for factor analysis of the C/E scale at week 2, as suggested by a Kaiser‐Meyer‐Olkin statistic of 0.84 and a significant Bartlett's test (*p* < .001). Based on visual inspection of the scree plot, the scale was clearly unifactorial, and all factor loadings were ≥0.46. We therefore proceeded to analyse credibility/expectancy as one composite construct (scale sum).

### Descriptive statistics pertaining to key process variables

All participants completed the pre‐treatment assessment. An overview of key variables used for the present study is provided in Table 2. Pearson correlations are also listed in Table 3. The correlations between credibility/expectancy and pre‐treatment health anxiety were −0.22 and −0.18, and correlations between the strength of the working alliance and pre‐treatment health anxiety were −0.05 and −0.04.

**TABLE 2:** Overview of key variables before imputation.

Variable	Measure	Time point	Total sample (*N* = 204)				ICBT (*n* = 102)			FTF‐CBT (*n* = 102)		
			*n*	Mean	SD	Median	*n*	Mean	SD	*n*	Mean	SD
Credibility/expectancy	C/E scale^a^	Week 2	199	36.4	7.6	37	100	35.3	8.4	99	37.5	6.6
Credibility/expectancy	C/E scale^a^	Week 8	185	37.7	8.0	39	90	37.8	8.0	95	37.7	8.0
Working alliance	WAI^b^	Week 2	199	34.3	7.3	36	100	32.3	7.9	99	36.3	5.9
Working alliance	WAI^b^	Week 8	185	35.4	6.7	36	90	34.6	6.9	95	36.1	6.5
Health anxiety	HAI‐18^c^	Week 0	204	34.0	6.4	35	102	33.9	6.5	102	34.2	6.4
Health anxiety	HAI‐18^c^	Week 1	196	32.1	6.8	32	101	32.5	6.6	95	31.7	7.1
Health anxiety	HAI‐18^c^	Week 2	193	30.6	7.2	30	96	30.8	7.2	97	30.5	7.2
Health anxiety	HAI‐18^c^	Week 3	184	29.1	7.8	29	94	29.2	7.7	90	29.1	7.9
Health anxiety	HAI‐18^c^	Week 4	184	27.8	8.1	27	90	27.9	8.3	94	27.6	8.0
Health anxiety	HAI‐18^c^	Week 5	183	27.1	8.2	27	90	26.7	7.7	93	27.5	8.6
Health anxiety	HAI‐18^c^	Week 6	179	27.1	8.4	27	90	26.7	8.8	89	27.6	8.0
Health anxiety	HAI‐18^c^	Week 7	176	26.4	8.3	26	89	26.0	8.3	87	26.8	8.4
Health anxiety	HAI‐18^c^	Week 8	173	25.5	8.8	25	88	25.5	9.0	85	25.5	8.6
Health anxiety	HAI‐18^c^	Week 9	175	24.6	8.6	24	87	24.1	8.9	88	25.2	8.3
Health anxiety	HAI‐18^c^	Week 10	173	23.2	8.6	23	84	22.7	8.4	89	23.7	8.8
Health anxiety	HAI‐18^c^	Week 11	172	21.8	8.5	22	83	22.2	8.0	89	21.4	8.9
Health anxiety	HAI‐18^c^	Week 12	194	20.7	8.6	20	97	21.0	8.5	97	20.4	8.7

*Note*: These are conventional HAI‐18 means (as opposed to estimated means derived from the linear mixed effects regression model). In this table, ‘Week 0’ refers to the pre‐treatment assessment before week 1, and ‘Week 12’ the post‐treatment assessment after week 12. ‘ICBT’ refers to Internet‐delivered cognitive behaviour therapy, and ‘FTF‐CBT’ refers to face‐to‐face cognitive behaviour therapy.

^a^ C/E scale = Credibility/Expectancy scale.

^b^ WAI = Working Alliance Inventory.

^c^ HAI‐18 = 18‐item Health Anxiety Inventory.

**TABLE 3:** Pearson correlations between key variables before imputation.

	C/E scale^a^ w2	C/E scale^a^ w8	WAI^b^ w2	WAI^b^ w8	HAI‐18^c^ w0	ΔHAI‐18^c^ w0‐2	ΔHAI‐18^c^ w2‐8	ΔHAI‐18^c^ w8‐12
Process variables								
C/E scale^a^ w2 (*n* = 199)	—							
C/E scale^a^ w8 (*n* = 185)	0.62*	—						
WAI^b^ w2 (*n* = 199)	0.49*	0.40*	—					
WAI^b^ w8 (*n* = 185)	0.53*	0.66*	0.59*	—				
Health anxiety								
HAI‐18^c^ w0	−0.22*	−0.18*	−0.05	−0.04	—			
ΔHAI‐18^c^ w0‐2	0.38*	0.36*	0.07	0.20*	−0.40*	—		
ΔHAI‐18^c^ w2‐8	0.19*	0.37*	0.16*	0.30*	0.04	0.27*	—	
ΔHAI‐18^c^ w8‐12	0.04	0.04	0.09	0.10	0.20*	−0.16*	0.25*	—
ΔHAI‐18^c^ w0‐12	0.29*	0.41*	0.17*	0.33*	−0.04	0.49*	0.90*	0.50*

*Note*: All HAI‐18 variables, including the slopes (Δ) represent the fitted values from a piecewise linear mixed effects regression model with splines at week 2 and 8. In this table, ‘w0’ refers to the pre‐treatment assessment before week 1, and ‘w12’ refers to the post‐treatment assessment after week 12. Asterisks denote statistical significance (*p* < .05).

^a^ C/E scale = Credibility/Expectancy scale.

^b^ WAI = Working Alliance Inventory.

^c^ HAI‐18 = 18‐item Health Anxiety Inventory.

### Change in credibility/expectancy, the working alliance and health anxiety

**FIGURE 2:**
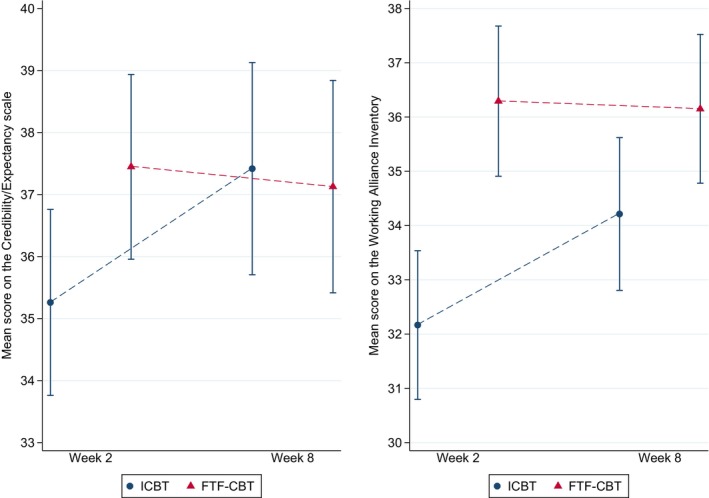
Mean credibility/expectancy and strength of the working alliance. Error bars denote 95% confidence intervals derived from the multiply imputed data. FTF‐CBT, face‐to‐face cognitive behaviour therapy; ICBT, Internet‐delivered cognitive behaviour therapy.

Change in mean credibility/expectancy and the strength of the working alliance is illustrated in Figure 2. Based on the multiply imputed data, credibility/expectancy was rated significantly lower in Internet‐delivered than in face‐to‐face CBT at week 2 (*t* = −2.05, *p* = .042; exactly .050 with non‐imputed data) but not at week 8 (*t* = 0.24, *p* = .811). Similarly, the strength of the working alliance was rated significantly lower in Internet‐delivered than in face‐to‐face CBT at week 2 (*t* = −4.17, *p* < .001) but not at week 8 (*t* = −1.92, *p* = .057). As was reported in the primary publication (Axelsson et al., 2020), both treatment groups lowered their HAI‐18 health anxiety mean score by 11.3 over the 12‐week treatment period (*p*s <0.001; within‐group *d*s = 1.76).

### Relationship between credibility/expectancy and the reduction in health anxiety

**FIGURE 3:**
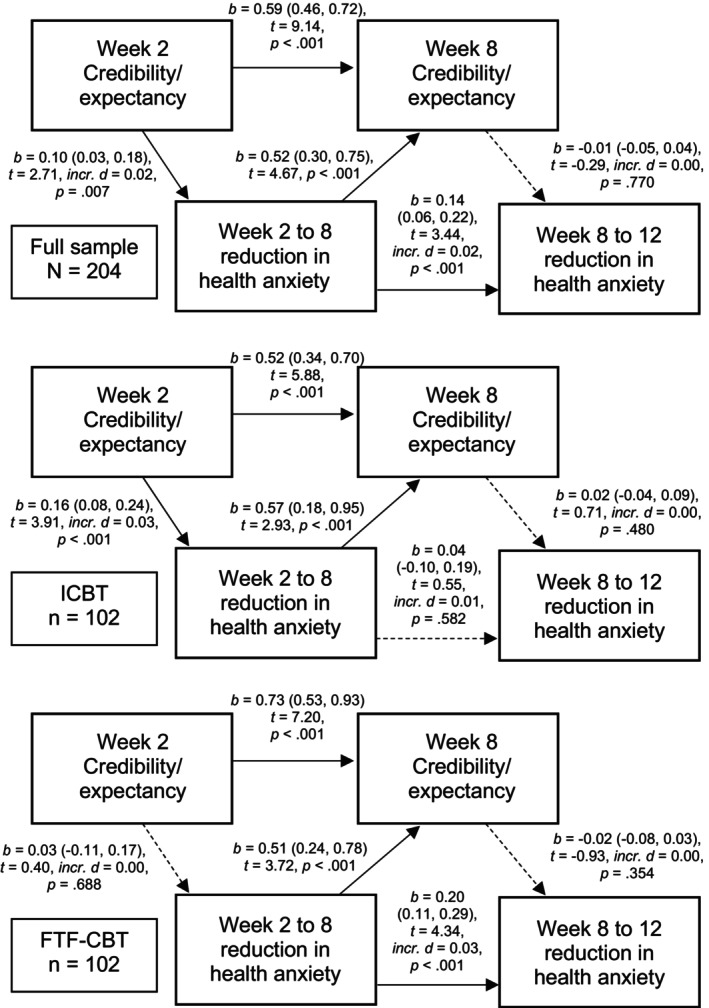
Regression paths pertaining to credibility/expectancy (the Credibility/Expectancy scale) in relation to change in health anxiety (the 18‐item Health Anxiety Inventory) over the course of cognitive behaviour therapy (CBT) for pathological health anxiety. These linear regression models were fitted on multiply imputed data and fitted regression lines derived from a linear mixed effects model that was run prior to the imputation (see the methods). One regression model was fitted per dependent variable. A higher anxiety reduction score was coded as being indicative of a larger reduction. Thus, for example, the score reduction from week 2 to week 8 was calculated as the week 2 score minus the week 8 score. Solid lines stand for statistical significance at α = 0.05. FTF‐CBT, face‐to‐face CBT; ICBT, therapist‐guided Internet‐delivered CBT.

The longitudinal relationship between credibility/expectancy and the reduction in health anxiety is illustrated in Figure 3. In the total sample and Internet‐delivered CBT, week 2 credibility/expectancy was significantly predictive of a larger subsequent symptom reduction, which was in turn predictive of a higher week 8 credibility/expectancy. Week 8 credibility/expectancy, however, was not significantly predictive of subsequent symptom reduction. In face‐to‐face CBT, week 2 to 8 symptom reduction was associated with a higher week 8 credibility/expectancy, but at no time point was credibility/expectancy a significant predictor of subsequent symptom reduction.

In auxiliary analyses, we performed direct tests of the treatment format (Internet‐delivered versus face‐to‐face CBT) as a moderator of the effect of credibility/expectancy on subsequent symptom reduction. The effect of week 2 credibility/expectancy on week 2 to 8 symptom reduction did not differ significantly between the treatment formats (*b* = 0.13 [95% CI –0.03, 0.29]). Similarly, the effect of week 8 credibility/expectancy on week 8 to 12 symptom reduction did not differ significantly between the treatment formats (*b* = 0.02 [95% CI –0.06, 0.09]).

### Relationship between the strength of the working alliance and the reduction in health anxiety

**FIGURE 4:**
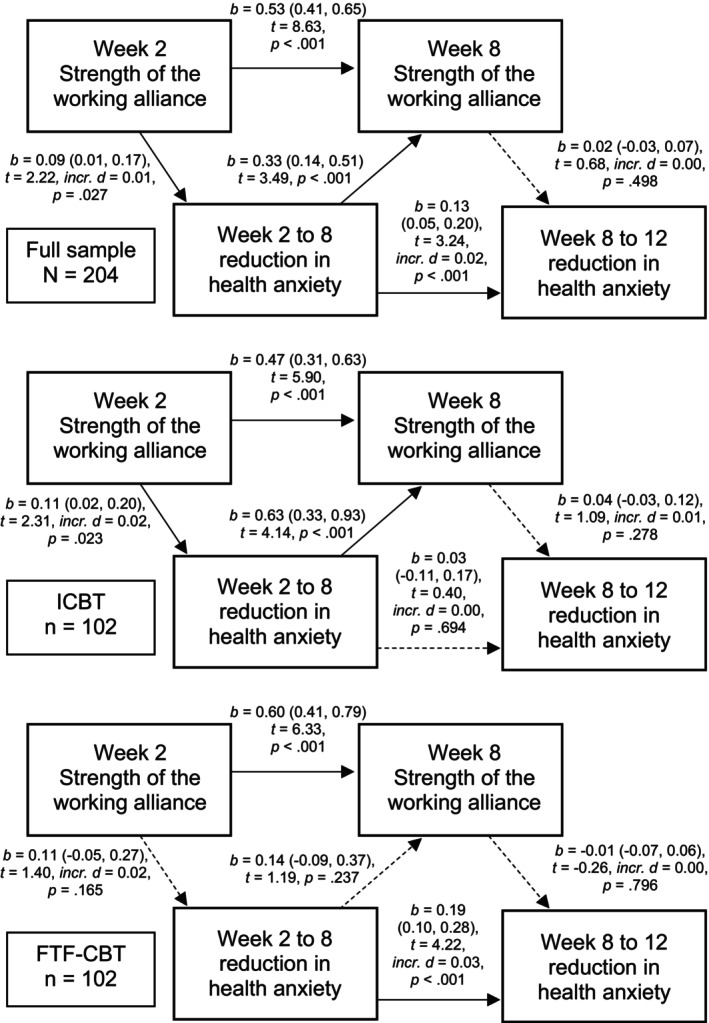
Regression paths pertaining to the strength of the working alliance (as rated by the patient, using the Working Alliance Inventory) in relation to change in health anxiety (the 18‐item Health Anxiety Inventory) over the course of cognitive behaviour therapy (CBT) for pathological health anxiety. These linear regression models were fitted on multiply imputed data and fitted regression lines derived from a linear mixed effects model that was run prior to the imputation (see the methods). One regression model was fitted per dependent variable. A higher anxiety reduction score was coded as being indicative of a larger reduction. Thus, for example, the score reduction from week 2 to week 8 was calculated as the week 2 score minus the week 8 score. Solid lines stand for statistical significance at α = 0.05. FTF‐CBT, face‐to‐face CBT; ICBT, therapist‐guided Internet‐delivered CBT.

The longitudinal relationship between the strength of the working alliance and the reduction in health anxiety is illustrated in Figure 4. In the total sample and Internet‐delivered CBT, a stronger week 2 working alliance was significantly predictive of a larger subsequent symptom reduction, which was in turn predictive of a stronger week 8 working alliance. The strength of the week 8 working alliance, however, was not significantly predictive of subsequent symptom reduction. In face‐to‐face CBT, there were no significant pathways between the strength of the working alliance and symptom reduction.

In auxiliary analyses, we performed direct tests of the treatment format (Internet‐delivered versus face‐to‐face CBT) as a moderator of the effect of the strength of the working alliance on subsequent symptom reduction. The effect of the week 2 working alliance on week 2 to 8 symptom reduction did not differ significantly between the treatment formats (*b* = −0.01 [95% CI –0.18, 0.17]). Similarly, the effect of the week 8 working alliance on week 8 to 12 symptom reduction did not differ significantly between the treatment formats (*b* = 0.02 [95% CI –0.08, 0.11]).

## DISCUSSION

This study investigated the associations between treatment credibility, the patient's expectancy of improvement, the strength of the working alliance and symptom reduction in cognitive behaviour therapy (CBT) for pathological health anxiety. This was based on data from a randomised controlled trial that compared Internet‐delivered and face‐to‐face CBT. Contrary to expectation, there was only mixed support for a reciprocal relationship between credibility/expectancy (analysed as one variable) or the strength of the working alliance, on the one hand, and the reduction in health anxiety on the other. Overall correlations between the process variables (credibility/expectancy, working alliance) and the 12‐week reduction in health anxiety were small to moderate, and slightly higher at week 8 than week 2 (Table 3). Week 2 credibility/expectancy and working alliance were significant predictors of subsequent symptom reduction. Week 8 credibility/expectancy and working alliance, however, appeared to be more closely related to previous ratings of the same domain and previous symptom reduction than subsequent symptom reduction (Table 3; Figure 3). In summary, as predictors, credibility/expectancy and the strength of the working alliance appear to be important primarily in the early stages of CBT. In contrast, credibility/expectancy and working alliance measurements at the latter stage of CBT are likely to say more about previous than subsequent symptom reduction.

### Differences over time in internet‐delivered versus face‐to‐face CBT

Because, to our knowledge, this study was based on the largest existing randomised direct comparison of Internet‐delivered and face‐to‐face CBT for an anxiety disorder, some further notes on similarities and differences with regard to these two delivery formats are warranted. At week two, both mean credibility/expectancy and the mean strength of the working alliance were significantly lower in Internet‐delivered than in face‐to‐face CBT. At week eight, the mean scores of the Internet‐delivered condition had increased so that there were no longer any significant differences versus the face‐to‐face format (Figure 2). It is perhaps not surprising that the participants were less convinced of the Internet‐delivered treatment in the initial phase of the trial, considering that this is a newer and less known treatment format. This said, the effect size was not very large, and in the existing literature, the typical (meta‐analytically pooled) finding has been that of similar credibility/expectancy levels in the two treatment formats, without a clear relationship with the time of administration (Pontén et al., 2024). Due to the small number of available studies, it is still too early to say to what degree this finding generalises over cultures, settings, psychiatric conditions, therapies and phases of treatment. In this particular study, the auxiliary direct tests did not indicate that the format moderated the effect of the process variables on subsequent symptom reduction.

### Potential causal interpretations

The fact that patient‐rated credibility/expectancy or working alliance is a predictor of subsequent reduction in health anxiety—or that the reduction in health anxiety is a predictor of subsequent credibility/expectancy or working alliance—does not necessarily mean that this reflects a causal relationship. In other words, it is not clear, for example, exactly why patients in CBT with a higher credibility/expectancy rating at week 2 subsequently reduce their health anxiety scores to a larger extent. One straightforward possibility is that those who perceive their treatment as more credible, and expect a more beneficial outcome, engage more with the treatment strategies (Beatty & Binnion, 2016), and perhaps also activate learned placebo responses, which results in more substantial symptom reduction. There is also room, however, for very different causal mechanisms. For example, patients with particular pre‐existing personal characteristics, such as a lower level of depression or more pro‐social personality traits, could, for this reason, be more inclined both to rate credibility/expectancy and the strength of the working alliance as higher, and to be able to improve more in their symptomatology. Another example of an alternative causal interpretation is that helpful therapist behaviours or a favourable psychosocial situation could explain both higher credibility/expectancy and working alliance ratings, and also a more pronounced reduction in health anxiety. Notably, it could also be the case that symptom reduction in the first two weeks of treatment was important for the process scores at week 2. Regardless of which mechanism is the primary driver behind the observed outcomes, this study lends support for the predictive role of early patient‐reported credibility/expectancy and working alliance in CBT for health anxiety. Future experimental manipulation of credibility/expectancy and the working alliance is warranted to understand this better.

### Strengths and limitations

Strengths of this study included the relatively large sample size, the use of repeated measurements and the high data retention. The primary limitation was that even though this trial was randomised to ensure experimental control of its primary outcome, the study of within‐group effects, such as those reported here, always implies a threat of confounding. Another limitation is that even though credibility/expectancy and the strength of the working alliance were measured at two time points, it would have been ideal if all variables had been measured over several points in time. This had made it possible to compare more specific phases of treatment with regard to the significance of potential processes. Another type of limitation is that we focused on openly reported, conscious, expectations only. Most probably, learned responses to psychotherapy need not be consciously realised in this manner (Kirsch et al., 2014). Future studies could be undertaken to determine if placebo‐like effects are better captured using experimental paradigms (Seewald et al., 2023). Similarly, the strength of the working alliance was only measured from the patient's point of view. It is possible that the outcome had been different, had the strength of the working alliance instead been assessed by the therapist. In support of this view, a previous study of CBT for pathological health anxiety found that patient and therapists ratings of the strength of the working alliance had a Pearson correlation of merely 0.47 (Weck et al., 2015). Last, we wish to emphasise that this was not a study of within‐individual change or minute changes, or deviations from the patient's typical functioning, from week to week. Rather, in this study, we focused on differences between individuals, and how these interact over time (Lüdtke & Robitzsch, 2023). In the language of Zilcha‐Mano and Fisher (2022), the type of analysis conducted here treats credibility/expectancy and the strength of the working alliance as relatively ‘trait‐like’ characteristics in the sense that we did not test for shorter‐term, for example week‐by‐week, relationships between fluctuations in the process variables and fluctuations in health anxiety. It is conceivable that process variables such as credibility/expectancy and the strength of the working alliance have both such shorter‐term, or ‘state‐like’, relationships with treatment outcomes, and more ‘trait‐like’ qualities perhaps more reflecting the personality of the patient and so on. Therefore, findings are likely to differ as a function of perspective.

### Clinical implications

The clinical implications of this study are primarily that credibility/expectancy and the strength of the working alliance appear to be important in the early stages of CBT. This lends tentative support for the common idea that it could be beneficial to select patients for CBT based on whether they perceive this treatment as credible and expectancy‐evoking, and also that there could be benefits of promoting expectancies before treatment. At the later stages of CBT, credibility/expectancy and working alliance measures are likely to say more about previous than subsequent symptom reduction. To the extent that larger effects are achieved over the middle phase of the treatment (from week 2 to 8), this is predictive of an improvement in the patient's view of credibility/expectancy later on.

### Implications for future research

This study indicates that the patient's view of credibility/expectancy and the strength of the working alliance is likely to change over the course of CBT for health anxiety. Future research aiming to study the role of credibility/expectancy and the working alliance would typically be advised to take this into consideration, either by focusing on the early phase of treatment or by incorporating repeated measurements over time.

## CONCLUSION

In CBT for health anxiety, the patient's early ratings of credibility/expectancy and the strength of the working alliance appear to be predictive of subsequent symptom reduction. The patient's later ratings of credibility/expectancy and the strength of the working alliance appear to be of more limited predictive utility.

## AUTHOR CONTRIBUTIONS

**Erland Axelsson:** Conceptualization; methodology; formal analysis; data curation; writing – original draft; writing – review and editing; investigation. **Erik Hedman‐Lagerlöf:** Writing – review and editing; investigation; methodology.

## CONFLICT OF INTEREST STATEMENT

EA and EHL have been involved in the development and evaluation of CBT for pathological health anxiety. EA and EHL have also authored books and book chapters on the topic of pathological health anxiety. EHL is a shareholder of Hedman‐Lagerlöf och Ljótsson psykologi AB: a company that licenses CBT manuals.

## Supporting information


Data S1.

## Data Availability

Individual participant data from this study fall under Swedish and European Union data protection and privacy legislation and can therefore not be shared freely. Requests will be considered on a case‐by‐case basis, so as to ensure that data and materials are managed in accordance with current legislation and the local policies of the sponsor.

## References

[papt12591-bib-0001] Aan de Stegge, B. M. , Tak, L. M. , Rosmalen, J. G. M. , & Oude Voshaar, R. C. (2018). Death anxiety and its association with hypochondriasis and medically unexplained symptoms: A systematic review. Journal of Psychosomatic Research, 115, 58–65. 10.1016/j.jpsychores.2018.10.002 30470318

[papt12591-bib-0002] Alberts, N. M. , Hadjistavropoulos, H. D. , Jones, S. L. , & Sharpe, D. (2013). The short health anxiety inventory: A systematic review and meta‐analysis. Journal of Anxiety Disorders, 27(1), 68–78. 10.1016/j.janxdis.2012.10.009 23247202

[papt12591-bib-0003] American Psychiatric Association . (2022). Diagnostic and statistical manual of mental disorders: DSM‐5‐TR. American Psychiatric Association.

[papt12591-bib-0004] Asmundson, G. J. G. , Taylor, S. , Carleton, R. N. , Weeks, J. W. , & Hadjstavropoulos, H. D. (2012). Should health anxiety be carved at the joint? A look at the health anxiety construct using factor mixture modeling in a non‐clinical sample. Journal of Anxiety Disorders, 26(1), 246–251. 10.1016/j.janxdis.2011.11.009 22169014

[papt12591-bib-0005] Axelsson, E. , Andersson, E. , Ljótsson, B. , Björkander, D. , Hedman‐Lagerlöf, M. , & Hedman‐Lagerlöf, E. (2020). Effect of internet vs face‐to‐face cognitive behavior therapy for health anxiety: A randomized noninferiority clinical trial. JAMA Psychiatry, 77(9), 915–924. 10.1001/jamapsychiatry.2020.0940 32401286 PMC7221860

[papt12591-bib-0006] Axelsson, E. , Andersson, E. , Ljótsson, B. , Wallhed Finn, D. , & Hedman, E. (2016). The health preoccupation diagnostic interview: Inter‐rater reliability of a structured interview for diagnostic assessment of DSM‐5 somatic symptom disorder and illness anxiety disorder. Cognitive Behaviour Therapy, 45(4), 259–269. 10.1080/16506073.2016.1161663 27096407

[papt12591-bib-0007] Axelsson, E. , & Hedman‐Lagerlöf, E. (2019). Cognitive behavior therapy for health anxiety: Systematic review and meta‐analysis of clinical efficacy and health economic outcomes. Expert Review of Pharmacoeconomics & Outcomes Research, 19(6), 663–676. 10.1080/14737167.2019.1703182 31859542

[papt12591-bib-0008] Axelsson, E. , & Hedman‐Lagerlöf, E. (2023a). Unwanted outcomes in cognitive behavior therapy for pathological health anxiety: A systematic review and a secondary original study of two randomized controlled trials. Expert Review of Pharmacoeconomics & Outcomes Research, 23(9), 1001–1015. 10.1080/14737167.2023.2250915 37614181

[papt12591-bib-0009] Axelsson, E. , & Hedman‐Lagerlöf, E. (2023b). Validity and clinical utility of distinguishing between DSM‐5 somatic symptom disorder and illness anxiety disorder in pathological health anxiety: Should we close the chapter? Journal of Psychosomatic Research, 165, 111133. 10.1016/j.jpsychores.2022.111133 36624001

[papt12591-bib-0010] Axelsson, E. , Österman, S. , & Hedman‐Lagerlöf, E. (2023). Joint factor analysis and approximate equipercentile linking of common trait health anxiety measures: A cross‐sectional study of the 14‐, 18‐ and 64‐item health anxiety inventory, the illness attitude scale, and the 14‐item Whiteley index. BMC Psychiatry, 23(1), 658. 10.1186/s12888-023-05151-7 37674135 PMC10483785

[papt12591-bib-0011] Baier, A. L. , Kline, A. C. , & Feeny, N. C. (2020). Therapeutic alliance as a mediator of change: A systematic review and evaluation of research. Clinical Psychology Review, 82, 101921. 10.1016/j.cpr.2020.101921 33069096

[papt12591-bib-0012] Beatty, L. , & Binnion, C. (2016). A systematic review of predictors of, and reasons for, adherence to online psychological interventions. International Journal of Behavioral Medicine, 23(6), 776–794. 10.1007/s12529-016-9556-9 26957109

[papt12591-bib-0013] Bordin, E. S. (1979). The generalizability of the psychoanalytic concept of the working alliance. Psychotherapy: Theory, Research & Practice, 16(3), 252–260. 10.1037/h0085885

[papt12591-bib-0014] Borkovec, T. D. , & Nau, S. D. (1972). Credibility of analogue therapy rationales. Journal of Behavior Therapy and Experimental Psychiatry, 3(4), 257–260. 10.1016/0005-7916(72)90045-6

[papt12591-bib-0015] Bressan, R. A. , Iacoponi, E. , de Candido Assis, J. , & Shergill, S. S. (2017). Hope is a therapeutic tool. BMJ, 359, j5469. 10.1136/bmj.j5469 29237595

[papt12591-bib-0016] Buchholz, J. L. , & Abramowitz, J. S. (2020). The therapeutic alliance in exposure therapy for anxiety‐related disorders: A critical review. Journal of Anxiety Disorders, 70, 102194. 10.1016/j.janxdis.2020.102194 32007734

[papt12591-bib-0017] Colloca, L. , & Miller, F. G. (2011). How placebo responses are formed: A learning perspective. Philosophical Transactions of the Royal Society of London. Series B: Biological Sciences, 366(1572), 1859–1869. 10.1098/rstb.2010.0398 21576143 PMC3130403

[papt12591-bib-0018] Cooper, K. , Gregory, J. D. , Walker, I. , Lambe, S. , & Salkovskis, P. M. (2017). Cognitive behaviour therapy for health anxiety: A systematic review and meta‐analysis. Behavioural and Cognitive Psychotherapy, 45(2), 110–123. 10.1017/S1352465816000527 28229805

[papt12591-bib-0019] Cuijpers, P. , Reijnders, M. , & Huibers, M. J. H. (2019). The role of common factors in psychotherapy outcomes. Annual Review of Clinical Psychology, 15, 207–231. 10.1146/annurev-clinpsy-050718-095424 30550721

[papt12591-bib-0020] Dimsdale, J. E. , Creed, F. , Escobar, J. , Sharpe, M. , Wulsin, L. , Barsky, A. , Lee, S. , Irwin, M. R. , & Levenson, J. (2013). Somatic symptom disorder: An important change in DSM. Journal of Psychosomatic Research, 75(3), 223–228. 10.1016/j.jpsychores.2013.06.033 23972410

[papt12591-bib-0021] DiNardo, P. A. , Brown, T. A. , & Barlow, D. H. (1994). Anxiety disorders interview schedule for DSM‐IV: Client interview schedule. Oxford University Press.

[papt12591-bib-0022] Elvins, R. , & Green, J. (2008). The conceptualization and measurement of therapeutic alliance: An empirical review. Clinical Psychology Review, 28(7), 1167–1187. 10.1016/j.cpr.2008.04.002 18538907

[papt12591-bib-0023] Ferguson, E. (2009). A taxometric analysis of health anxiety. Psychological Medicine, 39(2), 277–285. 10.1017/S0033291708003322 18485260

[papt12591-bib-0024] Fluckiger, C. , Del Re, A. C. , Wampold, B. E. , & Horvath, A. O. (2018). The alliance in adult psychotherapy: A meta‐analytic synthesis. Psychotherapy, 55(4), 316–340. 10.1037/pst0000172 29792475

[papt12591-bib-0025] Fluckiger, C. , Rubel, J. , Del Re, A. C. , Horvath, A. O. , Wampold, B. E. , Crits‐Christoph, P. , Atzil‐Slonim, D. , Compare, A. , Falkenstrom, F. , Ekeblad, A. , Errazuriz, P. , Fisher, H. , Hoffart, A. , Huppert, J. D. , Kivity, Y. , Kumar, M. , Lutz, W. , Muran, J. C. , Strunk, D. R. , … Barber, J. P. (2020). The reciprocal relationship between alliance and early treatment symptoms: A two‐stage individual participant data meta‐analysis. Journal of Consulting and Clinical Psychology, 88(9), 829–843. 10.1037/ccp0000594 32757587

[papt12591-bib-0026] Friedman, H. J. (1963). Patient‐expectancy and symptom reduction. Archives of General Psychiatry, 8(1), 61–67. 10.1001/archpsyc.1963.01720070063007 13963122

[papt12591-bib-0027] Furer, P. , & Walker, J. R. (2005). Treatment of hypochondriasis with exposure. Journal of Contemporary Psychotherapy, 35(3), 251–267. 10.1007/s10879-005-4319-y

[papt12591-bib-0028] Haller, K. , Becker, P. , Niemeyer, H. , & Boettcher, J. (2023). Who benefits from guided internet‐based interventions? A systematic review of predictors and moderators of treatment outcome. Internet Interventions, 33, 100635. 10.1016/j.invent.2023.100635 37449052 PMC10336165

[papt12591-bib-0029] Hatcher, R. L. , & Gillaspy, J. A. (2006). Development and validation of a revised short version of the working Alliance inventory. Psychotherapy Research, 16(1), 12–25. 10.1080/10503300500352500

[papt12591-bib-0030] Hedman, E. , Andersson, E. , Lekander, M. , & Ljótsson, B. (2015). Predictors in internet‐delivered cognitive behavior therapy and behavioral stress management for severe health anxiety. Behaviour Research and Therapy, 64, 49–55. 10.1016/j.brat.2014.11.009 25540862

[papt12591-bib-0031] Hedman, E. , Axelsson, E. , Andersson, E. , Lekander, M. , & Ljótsson, B. (2016). Exposure‐based cognitive‐behavioural therapy via the internet and as bibliotherapy for somatic symptom disorder and illness anxiety disorder: Randomised controlled trial. British Journal of Psychiatry, 209(5), 407–413. 10.1192/bjp.bp.116.181396 27491531

[papt12591-bib-0032] Hedman, E. , Axelsson, E. , Görling, A. , Ritzman, C. , Ronnheden, M. , El Alaoui, S. , Andersson, E. , Lekander, M. , & Ljótsson, B. (2014). Internet‐delivered exposure‐based cognitive‐behavioural therapy and behavioural stress management for severe health anxiety: Randomised controlled trial. British Journal of Psychiatry, 205(4), 307–314. 10.1192/bjp.bp.113.140913 25104835

[papt12591-bib-0033] Hedman‐Lagerlöf, E. , & Axelsson, E. (2019). Cognitive behavioral therapy for health anxiety. In E. Hedman‐Lagerlöf (Ed.), The Clinician's guide to treating health anxiety: Diagnosis, mechanisms, and effective treatment. Academic Press.

[papt12591-bib-0034] Höfling, V. , & Weck, F. (2013). Assessing bodily preoccupations is sufficient: Clinically effective screening for hypochondriasis. Journal of Psychosomatic Research, 75(6), 526–531. 10.1016/j.jpsychores.2013.10.011 24290041

[papt12591-bib-0035] Horvath, A. O. , Del Re, A. C. , Fluckiger, C. , & Symonds, D. (2011). Alliance in individual psychotherapy. Psychotherapy, 48(1), 9–16. 10.1037/a0022186 21401269

[papt12591-bib-0036] Horvath, A. O. , & Symonds, B. D. (1991). Relation between working alliance and outcome in psychotherapy: A meta‐analysis. Journal of Counseling Psychology, 38(2), 139–149. 10.1037/0022-0167.38.2.139

[papt12591-bib-0037] Kaiser, J. , Hanschmidt, F. , & Kersting, A. (2021). The association between therapeutic alliance and outcome in internet‐based psychological interventions: A meta‐analysis. Computers in Human Behavior, 114, 106512. 10.1016/j.chb.2020.106512

[papt12591-bib-0038] Kaptchuk, T. J. , & Miller, F. G. (2015). Placebo effects in medicine. New England Journal of Medicine, 373(1), 8–9. 10.1056/NEJMp1504023 26132938

[papt12591-bib-0039] Kazdin, A. E. (1979). Therapy outcome questions requiring control of credibility and treatment‐generated expectancies. Behavior Therapy, 10(1), 81–93. 10.1016/s0005-7894(79)80011-8

[papt12591-bib-0040] Kellner, R. (1986). Somatization and hypochondriasis. Praeger Publishers.

[papt12591-bib-0041] Kirsch, I. (2005). Placebo psychotherapy: Synonym or oxymoron? Journal of Clinical Psychology, 61(7), 791–803. 10.1002/jclp.20126 15827992

[papt12591-bib-0042] Kirsch, I. , Kong, J. , Sadler, P. , Spaeth, R. , Cook, A. , Kaptchuk, T. , & Gollub, R. (2014). Expectancy and conditioning in placebo analgesia: Separate or connected processes? Psychol Conscious, 1(1), 51–59. 10.1037/cns0000007 PMC4118664 25093194

[papt12591-bib-0043] Kumpasoğlu, G. B. , Campbell, C. , Saunders, R. , & Fonagy, P. (2024). Therapist and treatment credibility in treatment outcomes: A systematic review and meta‐analysis of clients' perceptions in individual and face‐to‐face psychotherapies. Psychotherapy Research, 35, 1–16. 10.1080/10503307.2023.2298000 38176020 PMC11771474

[papt12591-bib-0044] Leon, A. C. , Olfson, M. , Portera, L. , Farber, L. , & Sheehan, D. V. (1997). Assessing psychiatric impairment in primary care with the Sheehan disability scale. International Journal of Psychiatry in Medicine, 27(2), 93–105. 10.2190/t8em-c8yh-373n-1uwd 9565717

[papt12591-bib-0045] Longley, S. L. , Broman‐Fulks, J. J. , Calamari, J. E. , Noyes, R. , Wade, M. , & Orlando, C. M. (2010). A taxometric study of hypochondriasis symptoms. Behavior Therapy, 41(4), 505–514. 10.1016/j.beth.2010.02.002 21035614

[papt12591-bib-0046] Lüdtke, O. , & Robitzsch, A. (2023). A critique of the random intercept cross‐lagged panel model. PsyArXiv. 10.31234/osf.io/6f85c

[papt12591-bib-0047] Martin, D. J. , Garske, J. P. , & Davis, M. K. (2000). Relation of the therapeutic alliance with outcome and other variables: A meta‐analytic review. Journal of Consulting and Clinical Psychology, 68(3), 438–450. 10.1037/0022-006x.68.3.438 10883561

[papt12591-bib-0048] Mataix‐Cols, D. , Isomura, K. , Sidorchuk, A. , Rautio, D. , Ivanov, V. Z. , Ruck, C. , Osterman, S. , Lichtenstein, P. , Larsson, H. , Kuja‐Halkola, R. , Chang, Z. , Brickell, I. , Hedman‐Lagerlof, E. , & de la Fernandez Cruz, L. (2024). All‐cause and cause‐specific mortality among individuals with hypochondriasis. JAMA Psychiatry, 81(3), 284. 10.1001/jamapsychiatry.2023.4744 38091000 PMC10719832

[papt12591-bib-0049] Newby, J. M. , Hobbs, M. J. , Mahoney, A. E. J. , Wong, S. K. , & Andrews, G. (2017). DSM‐5 illness anxiety disorder and somatic symptom disorder: Comorbidity, correlates, and overlap with DSM‐IV hypochondriasis. Journal of Psychosomatic Research, 101, 31–37. 10.1016/j.jpsychores.2017.07.010 28867421

[papt12591-bib-0050] Newby, J. M. , Smith, J. , Uppal, S. , Mason, E. , Mahoney, A. E. , & Andrews, G. (2018). Internet‐based cognitive behavioral therapy versus psychoeducation control for illness anxiety disorder and somatic symptom disorder: A randomized controlled trial. Journal of Consulting and Clinical Psychology, 86(1), 89–98. 10.1037/ccp0000248 29172593

[papt12591-bib-0051] Österman, S. , Axelsson, E. , Lindefors, N. , Hedman‐Lagerlöf, E. , Hedman‐Lagerlöf, M. , Kern, D. , Svanborg, C. , & Ivanov, V. Z. (2022). The 14‐item short health anxiety inventory (SHAI‐14) used as a screening tool: Appropriate interpretation and diagnostic accuracy of the Swedish version. BMC Psychiatry, 22(1), 701. 10.1186/s12888-022-04367-3 36376898 PMC9664720

[papt12591-bib-0052] Pilowsky, I. (1967). Dimensions of hypochondriasis. British Journal of Psychiatry, 113(494), 89–93. 10.1192/bjp.113.494.89 6029373

[papt12591-bib-0053] Pontén, M. , Jonsjö, M. , Vadenmark, V. , Moberg, E. , Grannas, D. , Andersson, G. , Andrews, G. , Boersma, K. , Hedman‐Lagerlöf, E. , Kleinstaeuber, M. , Weise, C. , Kaldo, V. , Ljótsson, B. , Andersson, E. , Axelsson, E. , & Jensen, K. (2024). Association between expectations and clinical outcomes in online v. face‐to‐face therapy ‐ an individual participant data meta‐analysis. Psychological Medicine, 54(6), 1207–1214. 10.1017/S0033291723003033 37905404

[papt12591-bib-0054] Reiss, S. , Peterson, R. A. , Gursky, D. M. , & McNally, R. J. (1986). Anxiety sensitivity, anxiety frequency and the prediction of fearfulness. Behaviour Research and Therapy, 24(1), 1–8. 10.1016/0005-7967(86)90143-9 3947307

[papt12591-bib-0055] Salkovskis, P. M. , Rimes, K. , Warwick, H. , & Clark, D. (2002). The health anxiety inventory: Development and validation of scales for the measurement of health anxiety and hypochondriasis. Psychological Medicine, 32(5), 843–853. 10.1017/S0033291702005822 12171378

[papt12591-bib-0056] Schafer, J. L. , & Graham, J. W. (2002). Missing data: Our view of the state of the art. Psychological Methods, 7(2), 147–177. 10.1037/1082-989x.7.2.147 12090408

[papt12591-bib-0057] Schulz, K. F. , Altman, D. G. , & Moher, D. (2010). CONSORT 2010 statement: Updated guidelines for reporting parallel group randomised trials. BMC Medicine, 8(1), 1. 10.1186/1741-7015-8-18 20334633 PMC2860339

[papt12591-bib-0058] Seewald, A. , Teige‐Mocigemba, S. , & Rief, W. (2023). Outcome expectations in psychotherapy: Validation of the therapy single category implicit association test (therapy SC‐IAT). Cognitive Therapy and Research, 47(6), 894–908. 10.1007/s10608-023-10413-5

[papt12591-bib-0059] Sheehan, D. V. , Lecrubier, Y. , Sheehan, K. H. , Amorim, P. , Janavs, J. , Weiller, E. , Hergueta, T. , Baker, R. , & Dunbar, G. C. (1998). The mini‐international neuropsychiatric interview (M.I.N.I.): The development and validation of a structured diagnostic psychiatric interview for DSM‐IV and ICD‐10. Journal of Clinical Psychiatry, 59(Suppl 20), 22–33. https://www.ncbi.nlm.nih.gov/pubmed/9881538 9881538

[papt12591-bib-0060] Strappini, F. , Socci, V. , Saliani, A. M. , Grossi, G. , D'Ari, G. , Damato, T. , Pompili, N. , Alessandri, G. , & Mancini, F. (2022). The therapeutic alliance in cognitive‐behavioral therapy for obsessive‐compulsive disorder: A systematic review and meta‐analysis. Frontiers in Psychiatry, 13, 951925. 10.3389/fpsyt.2022.951925 36147968 PMC9488733

[papt12591-bib-0061] Sunderland, M. , Newby, J. M. , & Andrews, G. (2013). Health anxiety in Australia: Prevalence, comorbidity, disability and service use. British Journal of Psychiatry, 202(1), 56–61. 10.1192/bjp.bp.111.103960 22500013

[papt12591-bib-0062] Svanborg, P. , & Åsberg, M. (1994). A new self‐rating scale for depression and anxiety states based on the comprehensive psychopathological rating scale. Acta Psychiatrica Scandinavica, 89(1), 21–28. 10.1111/j.1600-0447.1994.tb01480.x 8140903

[papt12591-bib-0063] Wampold, B. E. , & Fluckiger, C. (2023). The alliance in mental health care: Conceptualization, evidence and clinical applications. World Psychiatry, 22(1), 25–41. 10.1002/wps.21035 36640398 PMC9840508

[papt12591-bib-0064] Weck, F. , Richtberg, S. , Jakob, M. , Neng, J. M. B. , & Höfling, V. (2015). Therapist competence and therapeutic alliance are important in the treatment of health anxiety (hypochondriasis). Psychiatry Research, 228(1), 53–58. 10.1016/j.psychres.2015.03.042 25977073

[papt12591-bib-0065] Weck, F. , Richtberg, S. , & Neng, J. (2014). Epidemiology of hypochondriasis and health anxiety: Comparison of different diagnostic criteria. Current Psychiatry Reviews, 10(1), 14–23. 10.2174/1573400509666131119004444

[papt12591-bib-0066] Zilcha‐Mano, S. , & Fisher, H. (2022). Distinct roles of state‐like and trait‐like patient–therapist alliance in psychotherapy. Nature Reviews Psychology, 1(4), 194–210. 10.1038/s44159-022-00029-z

