# Supplementary material for: Associations between treatment credibility, patient expectancies, working alliance and symptom trajectory in cognitive behaviour therapy for pathological health anxiety
Source: Psychol Psychother. 2025 Mar 27;98(3):779–98. doi: 10.1111/papt.12591 (PMC12346255; doi:10.1111/papt.12591)
Supplement: Supplementary file 1 — Data S1. [file PAPT-98-779-s001.pdf]

Supplement for: Associations between treatment credibility, patient expectancies, working alliance, and symptom trajectory in cognitive behaviour therapy for pathological health anxiety

HAI-18 piecewise regression model from which fitted lines were derived

Stata command, with AR1 covariance structure:

```
mixed hai18 time_w0to2 time_w2to8 time_w8to12 || participantid: time_w0to2 time_w2to8 time_w8to12, res(ar1, t(time))
```

Regression equation:

$hai18_{ij} = \beta 0 + \beta 1(time\_w0to2_{ij}) + \beta 2(time\_w2to8_{ij}) + \beta 3(time\_w8to12_{ij}) + [u0_j + u1_j(time\_w0to2_{ij}) + u2_j(time\_w2to8_{ij}) + u3_j(time\_w8to12_{ij})] + \varepsilon_{ij}$

- i represents the observation
- j represents the participant (i.e., participantid)
- square brackets denote random effects
- εij represents the residual error

Supplement for: Associations between treatment credibility, patient expectancies, working alliance, and symptom trajectory in cognitive behaviour therapy for pathological health anxiety

Script used for the analysis (with minor changes for improved readability)

[Stata 15.1]

```
// correlations between time subvariables ca 0.24-0.60
pwcorr time_w0to2 time_w2to8 time_w8to12

// compare fit without and with random effects of the time variables
quietly mixed hai18 time_w0to2 time_w2to8 time_w8to12 || participantid: , ml
est store model_1
estat ic
quietly mixed hai18 time_w0to2 time_w2to8 time_w8to12 || participantid: time_w0to2, ml
est store model_2
estat ic
quietly mixed hai18 time_w0to2 time_w2to8 time_w8to12 || participantid: time_w0to2 time_w2to8, ml
est store model_3
estat ic
quietly mixed hai18 time_w0to2 time_w2to8 time_w8to12 || participantid: time_w0to2 time_w2to8 time_w8to12, ml
est store model_4
estat ic

lrtest model_1 model_2
lrtest model_2 model_3
lrtest model_3 model_4

mixed hai18 time_w0to2 time_w2to8 time_w8to12 || participantid: time_w0to2 time_w2to8 time_w8to12, res(ar1, t(time))
predict hai18_fittedval, fitted

// convert to wide format
drop time_* time_* rownumber
reshape wide hai18 hai14 hai18_fittedval, i(participantid) j(time)

// calculate deltas from fitted slopes
gen delta_hai18_w0t2 = hai18_fittedval0 - hai18_fittedval2
gen delta_hai18_w2t8 = hai18_fittedval2 - hai18_fittedval8
gen delta_hai18_w8t12 = hai18_fittedval8 - hai18_fittedval12

gen delta_hai18_w0t12 = hai18_fittedval0 - hai18_fittedval12

// sums
sum cscale_sum_w2 cscale_sum_w8 wai_w2 wai_w8 hai18_fittedval0 delta_hai18_w0t2 delta_hai18_w2t8 delta_hai18_w8t12 delta_hai18_w0t12

// Pearson correlations
pwcorr cscale_sum_w2 cscale_sum_w8 wai_w2 wai_w8 hai18_fittedval0 delta_hai18_w0t2 delta_hai18_w2t8 delta_hai18_w8t12 delta_hai18_w0t12, sig
```

Supplement for: Associations between treatment credibility, patient expectancies, working alliance, and symptom trajectory in cognitive behaviour therapy for pathological health anxiety

[R 4.3.2 with mice 3.16.0 and nlme 3.1-164]

```
library(dplyr)
library(lavaan)
library(lavaanPlot)
library(mice)
library(miceadds)
library(moments)
library(nlme)
library(readxl)
library(semPlot)
library(semTools)

## cscale and wai, centered
hani_cewaiv2_final$c_cscale_sum_w2 <- scale(hani_cewaiv2_final$cscale_sum_w2, scale=FALSE)
hani_cewaiv2_final$c_wai_w2 <- scale(hani_cewaiv2_final$wai_w2, scale=FALSE)
hani_cewaiv2_final$c_cscale_sum_w8 <- scale(hani_cewaiv2_final$cscale_sum_w8, scale=FALSE)
hani_cewaiv2_final$c_wai_w8 <- scale(hani_cewaiv2_final$wai_w8, scale=FALSE)

## interactions if needed later, based on centered
hani_cewaiv2_final$c_icbtce_w2 <- hani_cewaiv2_final$icbt * hani_cewaiv2_final$c_cscale_sum_w2
hani_cewaiv2_final$c_icbtwai_w2 <- hani_cewaiv2_final$icbt * hani_cewaiv2_final$c_wai_w2
hani_cewaiv2_final$c_icbtce_w8 <- hani_cewaiv2_final$icbt * hani_cewaiv2_final$c_cscale_sum_w8
hani_cewaiv2_final$c_icbtwai_w8 <- hani_cewaiv2_final$icbt * hani_cewaiv2_final$c_wai_w8

## covariates if needed later, centered
hani_cewaiv2_final$c_edu_postsec <- scale(hani_cewaiv2_final$education_postsecondary, scale=FALSE)
hani_cewaiv2_final$c_fitted_bl_hai18 <- scale(hani_cewaiv2_final$shai18_finfit_w0, scale=FALSE)
hani_cewaiv2_final$c_bl_madrss <- scale(hani_cewaiv2_final$madrs_w0, scale=FALSE)
hani_cewaiv2_final$c_bl_asi <- scale(hani_cewaiv2_final$asi_w0, scale=FALSE)

########################################

# descriptives based on non-imputed data:

nrow(hani_cewaiv2_final[!is.na(hani_cewaiv2_final$cscale_sum_w2),])
mean(hani_cewaiv2_final$cscale_sum_w2, na.rm=TRUE)
sd(hani_cewaiv2_final$cscale_sum_w2, na.rm=TRUE)
median(hani_cewaiv2_final$cscale_sum_w2, na.rm=TRUE)
skewness(hani_cewaiv2_final$cscale_sum_w2, na.rm=TRUE)
kurtosis(hani_cewaiv2_final$cscale_sum_w2, na.rm=TRUE)

nrow(hani_cewaiv2_final[!is.na(hani_cewaiv2_final$cscale_sum_w8),])
mean(hani_cewaiv2_final$cscale_sum_w8, na.rm=TRUE)
sd(hani_cewaiv2_final$cscale_sum_w8, na.rm=TRUE)
median(hani_cewaiv2_final$cscale_sum_w8, na.rm=TRUE)
skewness(hani_cewaiv2_final$cscale_sum_w8, na.rm=TRUE)
```

```
kurtosis(hani_cewaiv2_final$cscale_sum_w8, na.rm=TRUE)

nrow(hani_cewaiv2_final[!is.na(hani_cewaiv2_final$wai_w2),])
mean(hani_cewaiv2_final$wai_w2, na.rm=TRUE)
sd(hani_cewaiv2_final$wai_w2, na.rm=TRUE)
median(hani_cewaiv2_final$wai_w2, na.rm=TRUE)
skewness(hani_cewaiv2_final$wai_w2, na.rm=TRUE)
kurtosis(hani_cewaiv2_final$wai_w2, na.rm=TRUE)

nrow(hani_cewaiv2_final[!is.na(hani_cewaiv2_final$wai_w8),])
mean(hani_cewaiv2_final$wai_w8, na.rm=TRUE)
sd(hani_cewaiv2_final$wai_w8, na.rm=TRUE)
median(hani_cewaiv2_final$wai_w8, na.rm=TRUE)
skewness(hani_cewaiv2_final$wai_w8, na.rm=TRUE)
kurtosis(hani_cewaiv2_final$wai_w8, na.rm=TRUE)

######################################
## prepare imputation

hani_cewaiv2_imp <- hani_cewaiv2_final %>%
 select(
   deltagarid_num,
   icbt,
   ftf,
   delta_hai18_w0t2,
   delta_hai18_w2t8,
   delta_hai18_w8t12,
   cscale_sum_w2,
   cscale_sum_w8,
   wai_w2,
   wai_w8,
   shai18_w0,
   sds_w0,
   madrs_w0,
   age,
   female,
   adm,
   employed_arbetar,
   dur_total_years,
   klin_pats_preferens,
   current_som_disease_ooto,
   education_postsecondary,
   samtalochmodulerstartade,
   c_icbtce_w2,
   c_icbtce_w8,
   c_icbtwai_w2,
   c_icbtwai_w8,
```

```
    c_edu_postsec,
    c_fitted_bl_hai18,
    c_bl_madrss,
    c_bl_asi
  )

# ensure that key variables are numeric
hani_cewaiv2_imp <- hani_cewaiv2_imp %>%
  mutate(
    deltagarid_num = as.numeric(deltagarid_num),
    icbt = as.numeric(icbt),
    ftf = as.numeric(ftf),
    delta_hai18_w0t2 = as.numeric(delta_hai18_w0t2),
    delta_hai18_w2t8 = as.numeric(delta_hai18_w2t8),
    delta_hai18_w8t12 = as.numeric(delta_hai18_w8t12),
    cscale_sum_w2 = as.numeric(cscale_sum_w2),
    cscale_sum_w8 = as.numeric(cscale_sum_w8),
    wai_w2 = as.numeric(wai_w2),
    wai_w8 = as.numeric(wai_w8),
    shai18_w0 = as.numeric(shai18_w0),
    sds_w0 = as.numeric(sds_w0),
    madrs_w0 = as.numeric(madrs_w0),
    age = as.numeric(age),
    female = as.numeric(female),
    adm = as.numeric(adm),
    employed_arbetar = as.numeric(employed_arbetar),
    dur_total_years = as.numeric(dur_total_years),
    klin_pats_preferens = as.numeric(klin_pats_preferens),
    current_som_disease_ooto = as.numeric(current_som_disease_ooto),
    education_postsecondary = as.numeric(education_postsecondary),
    samtalochmodulerstartade = as.numeric(samtalochmodulerstartade),
    c_icbtce_w2 = as.numeric(c_icbtce_w2),
    c_icbtce_w8 = as.numeric(c_icbtce_w8),
    c_icbtwai_w2 = as.numeric(c_icbtwai_w2),
    c_icbtwai_w8 = as.numeric(c_icbtwai_w8),
    c_edu_postsec = as.numeric(c_edu_postsec),
    c_fitted_bl_hai18 = as.numeric(c_fitted_bl_hai18),
    c_bl_madrss = as.numeric(c_bl_madrss),
    c_bl_asi = as.numeric(c_bl_asi)
  )

# select relevant baseline predictors
hani_cewaiv2_imp_w0d <- hani_cewaiv2_imp %>%
  select(
    "delta_hai18_w0t2",
    "delta_hai18_w2t8",
    "delta_hai18_w8t12",
```

```
  "cscale_sum_w2",
  "cscale_sum_w8",
  "wai_w2",
  "wai_w8",
  "shai18_w0",
  "sds_w0",
  "madrs_w0",
  "age",
  "female",
  "adm",
  "employed_arbetar",
  "dur_total_years",
  "klin_pats_preferens",
  "current_som_disease_ooto",
  "education_postsecondary",
  "samtalochmodulerstartade",
  "c_icbtce_w2",
  "c_icbtce_w8",
  "c_icbtwai_w2",
  "c_icbtwai_w8"
 )

cor <- cor(hani_cewaiv2_imp_w0d, method = "pearson", use = "complete.obs")
cor_dataframe <- as.data.frame(cor)
# round all correlations to 2 decimals
cor_dataframe <- round(cor_dataframe, 2)

######################################
## perform imputation

## initial mice object, to extract method and predictor matrix to fill w values
ini <- mice(hani_cewaiv2_imp, maxit = 0)

# indicate variables to impute (by group)
meth <- ini$meth
meth["delta_hai18_w0t2"] <- "bygroup"
meth["delta_hai18_w2t8"] <- "bygroup"
meth["delta_hai18_w8t12"] <- "bygroup"
meth["cscale_sum_w2"] <- "bygroup"
meth["cscale_sum_w8"] <- "bygroup"
meth["wai_w2"] <- "bygroup"
meth["wai_w8"] <- "bygroup"
meth["shai18_w0"] <- "bygroup"
meth["sds_w0"] <- "bygroup"
meth["madrs_w0"] <- "bygroup"
meth["age"] <- "bygroup"
meth["female"] <- "bygroup"
```

```
meth["adm"] <- "bygroup"
meth["employed_arbetar"] <- "bygroup"
meth["dur_total_years"] <- "bygroup"
meth["klin_pats_preferens"] <- "bygroup"
meth["current_som_disease_ooto"] <- "bygroup"
meth["education_postsecondary"] <- "bygroup"
meth["samtalochmodulerstartade"] <- "bygroup"
meth["c_icbtce_w2"] <- "bygroup"
meth["c_icbtce_w8"] <- "bygroup"
meth["c_icbtwai_w2"] <- "bygroup"
meth["c_icbtwai_w8"] <- "bygroup"
meth

group <- list(
  "delta_hai18_w0t2" = "icbt",
  "delta_hai18_w2t8" = "icbt",
  "delta_hai18_w8t12" = "icbt",
  "cscale_sum_w2" = "icbt",
  "cscale_sum_w8" = "icbt",
  "wai_w2" = "icbt",
  "wai_w8" = "icbt",
  "shai18_w0" = "icbt",
  "sds_w0" = "icbt",
  "madrs_w0" = "icbt",
  "age" = "icbt",
  "female" = "icbt",
  "adm" = "icbt",
  "employed_arbetar" = "icbt",
  "dur_total_years" = "icbt",
  "klin_pats_preferens" = "icbt",
  "current_som_disease_ooto" = "icbt",
  "education_postsecondary" = "icbt",
  "samtalochmodulerstartade" = "icbt",
  "c_icbtce_w2" = "icbt",
  "c_icbtce_w8" = "icbt",
  "c_icbtwai_w2" = "icbt",
  "c_icbtwai_w8" = "icbt"
)

impfunction <- list(
  "delta_hai18_w0t2" = "pmm",
  "delta_hai18_w2t8" = "pmm",
  "delta_hai18_w8t12" = "pmm",
  "cscale_sum_w2" = "pmm",
  "cscale_sum_w8" = "pmm",
  "wai_w2" = "pmm",
  "wai_w8" = "pmm",
```

```
  "shai18_w0" = "pmm",
  "sds_w0" = "pmm",
  "madrs_w0" = "pmm",
  "age" = "pmm",
  "female" = "pmm",
  "adm" = "pmm",
  "employed_arbetar" = "pmm",
  "dur_total_years" = "pmm",
  "klin_pats_preferens" = "pmm",
  "current_som_disease_ooto" = "pmm",
  "education_postsecondary" = "pmm",
  "samtalochmodulerstartade" = "pmm",
  "c_icbtce_w2" = "pmm",
  "c_icbtce_w8" = "pmm",
  "c_icbtwai_w2" = "pmm",
  "c_icbtwai_w8" = "pmm"
)

# extract and fix predictor matrix
pred <- ini$pred
pred[ , ] <- 0
# participantid cluster variable (-2)
pred[ ,"deltagarid_num"] <- -2
# predictors are defined
pred[ ,"cscale_sum_w2"] <- 1
pred[ ,"wai_w2"] <- 1
pred[ ,"shai18_w0"] <- 1
pred[ ,"sds_w0"] <- 1
pred[ ,"madrs_w0"] <- 1
pred[ ,"age"] <- 1
pred[ ,"female"] <- 1
pred[ ,"adm"] <- 1
pred[ ,"employed_arbetar"] <- 1
pred[ ,"dur_total_years"] <- 1
pred[ ,"klin_pats_preferens"] <- 1
pred[ ,"current_som_disease_ooto"] <- 1
pred[ ,"education_postsecondary"] <- 1
pred[ ,"samtalochmodulerstartade"] <- 1
# week 2 should not predict each other
pred["cscale_sum_w2","wai_w2"] <- 0
pred["wai_w2","cscale_sum_w2"] <- 0
# null auto prediction
pred["delta_hai18_w0t2","delta_hai18_w0t2"] <- 0
pred["delta_hai18_w2t8","delta_hai18_w2t8"] <- 0
pred["delta_hai18_w8t12","delta_hai18_w8t12"] <- 0
pred["cscale_sum_w2","cscale_sum_w2"] <- 0
pred["cscale_sum_w8","cscale_sum_w8"] <- 0
```

```
pred["wai_w2","wai_w2"] <- 0
pred["wai_w8","wai_w8"] <- 0
pred["shai18_w0","shai18_w0"] <- 0
pred["sds_w0","sds_w0"] <- 0
pred["madrs_w0","madrs_w0"] <- 0
pred["age","age"] <- 0
pred["female","female"] <- 0
pred["adm","adm"] <- 0
pred["employed_arbetar","employed_arbetar"] <- 0
pred["dur_total_years","dur_total_years"] <- 0
pred["klin_pats_preferens","klin_pats_preferens"] <- 0
pred["current_som_disease_ooto","current_som_disease_ooto"] <- 0
pred["education_postsecondary","education_postsecondary"] <- 0
pred["samtalochmodulerstartade","samtalochmodulerstartade"] <- 0
# View(pred)

## imputation
impobject_hani_cewaiv2_final <- mice(hani_cewaiv2_imp, method = meth, predictorMatrix = pred,
                         m = 20, maxit = 20, group = group, imputationFunction = impfunction, seed = 12345)

## write imputed data to file
saveRDS(impobject_hani_cewaiv2_final, "[PATH]")

## read imputed data
read_impobject_hani_cewaiv2_final <- readRDS("[PATH]")

## ICBT vs. FTF-CBT subsets of MI-imputed data
impobject_hani_cewaiv2_final.icbt <- filter(read_impobject_hani_cewaiv2_final, icbt == 1)
impobject_hani_cewaiv2_final.ftf <- filter(read_impobject_hani_cewaiv2_final, icbt == 0)


########################################
## Change in credibility/expectancy (C/E), the working alliance (WAI), and health anxiety (HAI-18)
########################################

## ICBT vs. FTF-CBT t-tests

options(digits=4)

fitv2.t.test.ce <- with(data=read_impobject_hani_cewaiv2_final, exp=lm(cscale_sum_w2 ~ icbt))
t.test.estimates.ce <- pool(fitv2.t.test.ce)
summary(t.test.estimates.ce, conf.int=TRUE)
fitv2.t.test.ce.inv <- with(data=read_impobject_hani_cewaiv2_final, exp=lm(cscale_sum_w2 ~ ftf))
t.test.estimates.ce.inv <- pool(fitv2.t.test.ce.inv)
summary(t.test.estimates.ce.inv, conf.int=TRUE)

fitv8.t.test.ce <- with(data=read_impobject_hani_cewaiv2_final, exp=lm(cscale_sum_w8 ~ icbt))
```

Supplement for: Associations between treatment credibility, patient expectancies, working alliance, and symptom trajectory in cognitive behaviour therapy for pathological health anxiety

```
t.test.estimates.ce <- pool(fitv8.t.test.ce)
summary(t.test.estimates.ce, conf.int=TRUE)
fitv8.t.test.ce.inv <- with(data=read_impobject_hani_cewaiv2_final, exp=lm(cscale_sum_w8 ~ ftf))
t.test.estimates.ce.inv <- pool(fitv8.t.test.ce.inv)
summary(t.test.estimates.ce.inv, conf.int=TRUE)

fitv2.t.test.wai <- with(data=read_impobject_hani_cewaiv2_final, exp=lm(wai_w2 ~ icbt))
t.test.estimates.wai <- pool(fitv2.t.test.wai)
summary(t.test.estimates.wai, conf.int=TRUE)
fitv2.t.test.wai.inv <- with(data=read_impobject_hani_cewaiv2_final, exp=lm(wai_w2 ~ ftf))
t.test.estimates.wai.inv <- pool(fitv2.t.test.wai.inv)
summary(t.test.estimates.wai.inv, conf.int=TRUE)

fitv8.t.test.wai <- with(data=read_impobject_hani_cewaiv2_final, exp=lm(wai_w8 ~ icbt))
t.test.estimates.wai <- pool(fitv8.t.test.wai)
summary(t.test.estimates.wai, conf.int=TRUE)
fitv8.t.test.wai.inv <- with(data=read_impobject_hani_cewaiv2_final, exp=lm(wai_w8 ~ ftf))
t.test.estimates.wai.inv <- pool(fitv8.t.test.wai.inv)
summary(t.test.estimates.wai.inv, conf.int=TRUE)


########################################
## Analysis of the credibility/expectancy (C/E) and the working alliance (WAI) in relation to health anxiety (HAI-18)
########################################


### C/E scale vs. HAI-18 over time
options(digits = 4)
summary(pool(with(read_impobject_hani_cewaiv2_final, lm(delta_hai18_w2t8 ~ cscale_sum_w2))), conf.int = TRUE)
summary(pool(with(read_impobject_hani_cewaiv2_final, lm(cscale_sum_w8 ~ cscale_sum_w2 + delta_hai18_w2t8))), conf.int = TRUE)
summary(pool(with(read_impobject_hani_cewaiv2_final, lm(delta_hai18_w8t12 ~ cscale_sum_w8 + delta_hai18_w2t8))), conf.int = TRUE)

# Direct tests of ICBT vs FTF as a moderator of the effect of C/E on symptom reduction:
summary(pool(with(read_impobject_hani_cewaiv2_final, lm(delta_hai18_w2t8 ~ cscale_sum_w2 + icbt + cscale_sum_w2*icbt))), conf.int = TRUE)
summary(pool(with(read_impobject_hani_cewaiv2_final, lm(delta_hai18_w8t12 ~ cscale_sum_w8 + delta_hai18_w2t8 + icbt + cscale_sum_w8*icbt))), conf.int = TRUE)


### WAI vs. HAI-18 over time
summary(pool(with(read_impobject_hani_cewaiv2_final, lm(delta_hai18_w2t8 ~ wai_w2))), conf.int = TRUE)
summary(pool(with(read_impobject_hani_cewaiv2_final, lm(wai_w8 ~ wai_w2 + delta_hai18_w2t8))), conf.int = TRUE)
summary(pool(with(read_impobject_hani_cewaiv2_final, lm(delta_hai18_w8t12 ~ wai_w8 + delta_hai18_w2t8))), conf.int = TRUE)

# Direct tests of ICBT vs FTF as a moderator of the effect of WAI on symptom reduction:
summary(pool(with(read_impobject_hani_cewaiv2_final, lm(delta_hai18_w2t8 ~ wai_w2 + icbt + wai_w2*icbt))), conf.int = TRUE)
summary(pool(with(read_impobject_hani_cewaiv2_final, lm(delta_hai18_w8t12 ~ wai_w8 + delta_hai18_w2t8 + icbt + wai_w8*icbt))), conf.int = TRUE)
```

Supplement for: Associations between treatment credibility, patient expectancies, working alliance, and symptom trajectory in cognitive behaviour therapy for pathological health anxiety

```
######################################
## ICBT ONLY

### C/E scale vs. HAI-18 over time
summary(pool(with(impobject_hani_cewaiv2_final.icbt, lm(delta_hai18_w2t8 ~ cscale_sum_w2))), conf.int = TRUE)
summary(pool(with(impobject_hani_cewaiv2_final.icbt, lm(cscale_sum_w8 ~ cscale_sum_w2 + delta_hai18_w2t8))), conf.int = TRUE)
summary(pool(with(impobject_hani_cewaiv2_final.icbt, lm(delta_hai18_w8t12 ~ cscale_sum_w8 + delta_hai18_w2t8))), conf.int = TRUE)

### WAI vs. HAI-18 over time
# in the following, "statistic" stands for t
summary(pool(with(impobject_hani_cewaiv2_final.icbt, lm(delta_hai18_w2t8 ~ wai_w2))), conf.int = TRUE)
summary(pool(with(impobject_hani_cewaiv2_final.icbt, lm(wai_w8 ~ wai_w2 + delta_hai18_w2t8))), conf.int = TRUE)
summary(pool(with(impobject_hani_cewaiv2_final.icbt, lm(delta_hai18_w8t12 ~ wai_w8 + delta_hai18_w2t8))), conf.int = TRUE)


######################################
## FTF-CBT ONLY

### C/E scale vs. HAI-18 over time
summary(pool(with(impobject_hani_cewaiv2_final.ftf, lm(delta_hai18_w2t8 ~ cscale_sum_w2))), conf.int = TRUE)
summary(pool(with(impobject_hani_cewaiv2_final.ftf, lm(cscale_sum_w8 ~ cscale_sum_w2 + delta_hai18_w2t8))), conf.int = TRUE)
summary(pool(with(impobject_hani_cewaiv2_final.ftf, lm(delta_hai18_w8t12 ~ cscale_sum_w8 + delta_hai18_w2t8))), conf.int = TRUE)

### WAI vs. HAI-18 over time
# in the following, "statistic" stands for t
summary(pool(with(impobject_hani_cewaiv2_final.ftf, lm(delta_hai18_w2t8 ~ wai_w2))), conf.int = TRUE)
summary(pool(with(impobject_hani_cewaiv2_final.ftf, lm(wai_w8 ~ wai_w2 + delta_hai18_w2t8))), conf.int = TRUE)
summary(pool(with(impobject_hani_cewaiv2_final.ftf, lm(delta_hai18_w8t12 ~ wai_w8 + delta_hai18_w2t8))), conf.int = TRUE)
```
